# How did Sweden Fail the Pandemic?

**DOI:** 10.1177/0020731421994848

**Published:** 2021-02-26

**Authors:** Finn Diderichsen

**Affiliations:** 1 4321 University of Copenhagen, Copenhagen, Denmark

**Keywords:** COVID-19, Sweden, policy, inequality

## Abstract

Sweden has since the start of the pandemic a COVID-19 mortality rate that is 4 to 10 times higher than in the other Nordic countries. Also, measured as age-standardized all-cause excess mortality in the first half of 2020 compared to previous years Sweden failed in comparison with the other Nordic countries, but only among the elderly. Sweden has large socioeconomic and ethnic inequalities in COVID-19 mortality. Geographical, ethnic, and socioeconomic inequalities in mortality can be due to differential exposure to the virus, differential immunity, and differential survival. Most of the country differences are due to differential exposure, but the socioeconomic disparities are mainly driven by differential survival due to an unequal burden of comorbidity. Sweden suffered from an unfortunate timing of tourists returning from virus hotspots in the Alps and Sweden's government response came later and was much more limited than elsewhere. The government had an explicit priority to protect the elderly in nursing and care homes but failed to do so. The staff in elderly care are less qualified and have harder working conditions in Sweden, and they lacked adequate care for the clients. Sweden has in recent years diverged from the Scandinavian welfare model by strong commercialization of primary care and elderly care.

## COVID-19 Burden in Sweden

Sweden has long been known for favorable levels of population health, with a life expectancy that since the 1970s is among the highest in Europe. The current development in 2020 seems to represent a significant change. Statistics Sweden predicted in late November 2020 that male life expectancy in 2020 would be 0.9 years lower than in 2019.^1^ This is a unique decline not seen since the H1N1 pandemic in 1918, where life expectancy declined 5.2 years compared to 1917.

In Table 1, we have in the left column shown the cumulative rate of deaths where COVID-19 is registered as the underlying or contributing cause.^2^ It is 4 to 10 times higher in Sweden than in the other Nordic countries, but lower than in the United Kingdom and Spain. The national COVID-19 death rates are not age standardized and are influenced by different test rates and death-certificate routines. In the two middle columns, we have therefore shown data from a study by the British Office of National Statistics^3^ on the age-standardized all-cause mortality in the first half of 2020 compared with the average 2015 to 2019. It shows that Spain and the United Kingdom had a substantial excess mortality in 2020. For Sweden, the excess mortality was lower and only found among the elderly, particularly among those living in nursing homes.^4,5^ The excess mortality rates are influenced not only by the mortality in 2020 but also by the period of comparison, that is, 2015 to 2019. All the countries mentioned in Table 1, in particular Spain and the United Kingdom, had a considerable excess mortality in the influenza season in 2018. The 2019 influenza was much milder, in particular in Sweden.^6^ COVID-19 survival has improved, but mortality rates now in the second wave (November-December) are still higher in Sweden compared to the other Nordic countries. What went wrong in Sweden and how?

**Table 1.** COVID-19 Mortality Rates and All-Cause Excess Mortality and GDP Change in Six European Countries During COVID-19 Pandemic.

	Cumulative COVID-deaths per 100 000 Jan.-Dec. 28, 2020^a^	Age-standardized excess all-cause mortality (%) in Jan.-June 2020 compared to 2015 to 2019^b^		Decline in GDP (%) in second quarter compared to First quarter of 2020^c^
		0-64 years	65+ years	
Sweden	84.3	−2.6	+2.9	−8.3
Norway	8.0	−3.6	−2.6	−5.1
Denmark	21.2	−6.4	−2.4	−6.8
Finland	9.9	−3.1	−2.2	−4.4
United Kingdom	105.5	+5.1	+7.3	−19.8
Spain	107.2	+2.6	+7.3	−17.8

^a^ Source: ECDC 2020^2^ (accessed December 16, 2020).

^b^ Source: ONS 2020.^3^ All-cause mortality is calculated as a percentage change in the age-standardized all-cause mortality rate in January to June 2020 compared to the average 2015 to 2019.

^c^ Source: OECD 2020.^7^

Abbreviation: GDP, gross domestic product.

## Vulnerable Groups in Sweden

Differences in mortality between population groups within Sweden might provide some guidance. A large population-based cohort study on all deaths during the first 3 months of the pandemic has estimated relative risks for COVID-19 deaths and all other causes of death.^8^ It shows substantial socioeconomic variations that are stronger for COVID-19 deaths than for the other causes (Table 2). There is a high mortality not only linked to higher age and male gender but also linked to less education and low income. COVID-19 mortality—but not mortality from other causes—is higher among immigrants from low- and middle-income countries (LMIC) than among the Swedish born, and among people living in Stockholm.

**Table 2.** Age-Standardized Hazard Ratios for Deaths in COVID-19 and All Other Causes in Sweden March 13 to May 7, 2020, *N* = 7,775,064.

	0 to 65 years		66 + years	
	COVID-19 death	All other causes	COVID-19 death	All other causes
Age 70+ years (ref. 20-49 years)			240.65*	82.29*
Men (ref. women)	2.97*	1.84*	2.01*	1.61*
Primary education (ref. post-secondary education)	2.62*	1.94*	1.30*	1.30*
Lowest income tercile (ref. highest tercile)	5.40*	4.00*	1.35*	1.45*
Migrant born in low- and middle-income countries (ref. born in Sweden)	2.39*	0.61*	2.15*	0.98
Living in Stockholm (ref. rest of the country)	3.86*	1.12	3.90*	1.16*

Source: Drefahl et al.^8^

*p < .001.

This means that there is a concentration of mortality to vulnerable groups: 91% of deaths occurred among those aged 70+ and 61% among the lowest income tercile. Despite higher age-adjusted mortality rates for immigrants from LMIC, only 11% of deaths occurred among this group.^8^

## Mechanisms Driving the Inequality

**Figure 1.**
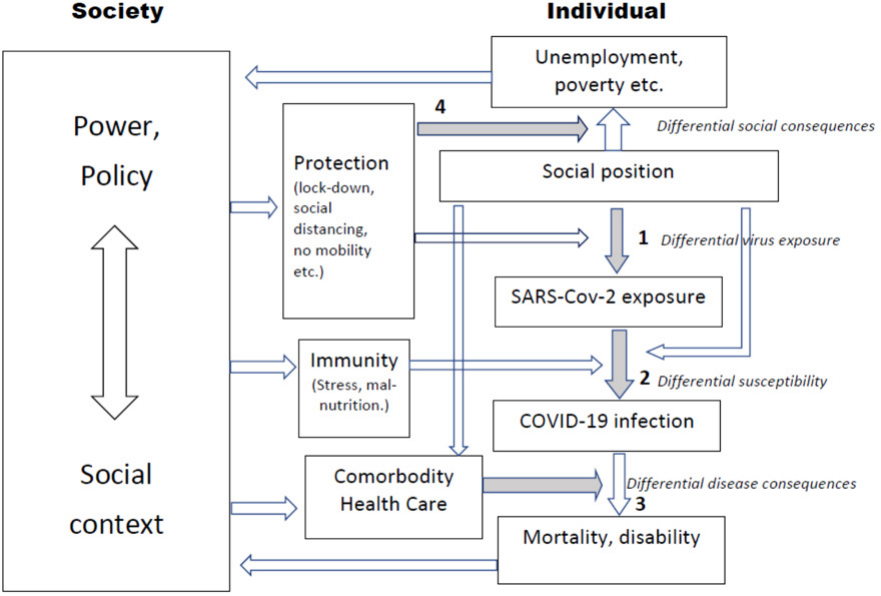
Model of causal pathways and policy entry points framing inequality of COVID-19 and its disease and social consequences. Modified after Diderichsen et al.^9,10^

Applying a model primarily used to analyze socioeconomic inequalities,^9,10^ 3 potential pathways can be identified (see Figure 1): (1) *differential exposure* to SARS-COVID-2 virus, (2) *differential susceptibility* to the health effects of the virus, and (3) *differential disease consequences* including mortality. The model also illustrates the (4) *differential social consequences* in terms of unemployment, poverty, etc, which may be the unintended result of policies implemented to protect the population from exposure. The model points out where policies and other types of contextual influences can modify the 4 mechanisms.

Both the country differences found in Table 1 and the sociodemographic inequalities found in Table 2 can in theory be driven by the 3 mechanisms (1-3) shown in Figure 1. Survival in COVID-19 is strongly related to comorbidity in a broad range of noncommunicable diseases including coronary heart disease, malignancies, kidney disease, obesity, diabetes, chronic obstructive pulmonary disease, and dementia.^11^ All these disorders occur with a social gradient in European countries, including Sweden as illustrated by the hazard ratios of “all other causes.” They might therefore contribute to the inequalities between educational groups and income levels in COVID-19 found in Table 2. They do not, however, seem to play any role in the ethnic inequalities, since the ratios for “all other causes” are actually lower among immigrants compared with the Swedish born.

*Differential susceptibility* will, in the case of viral disease mean differential immunity (2). Immunity is in December 2020 not yet influenced by any vaccine. The immune system might however play a major role in the strong influence of age and male gender. The steep social gradient in obesity and social stress might generate the social and ethnic inequalities in susceptibility to virus. Obesity weakens the immune response^12^ and several studies have shown an effect of stress.^13–15^

*Differential exposure* (1) generates some of the socioeconomic differences through work and housing environments. Employees working in jobs that involve close contact with the public and colleagues are at higher risk. Health and elderly care workers are particularly at risk as they are most likely to meet infected people. While many working in professional occupations have been able to work from home, this has not been an option for employees in health and elderly care, food production and distribution, transportation, security, etc. Swedish studies have identified taxi and bus drivers as occupations with high incidence^16^ and mortality.^17^ Occupational differences in mortality are otherwise found to be smaller in Sweden compared to what is found in the United Kingdom.^18^ Housing conditions are important, even in Sweden, which has relatively high housing standards. There are large variations in COVID-19 mortality across different neighborhoods in the Stockholm region.^4^ Deprived areas with high COVID-19 mortality have high proportions of low-income people and immigrants. After adjustment for compositional effects of individual education, income, and ethnicity substantial area differences remain.^19^ Housing density is higher and multigenerational households are more common in deprived areas and contribute to higher exposure of susceptible groups. Viral load influences not only incidence but also survival.^20^ Swedish studies indicate that for persons aged 70+ years sharing a household with someone of working age and living in a densely populated area means a higher death risk.^4^

The international differences in mortality shown in Table 1 are only to a very limited degree explained by differences in susceptibility and comorbidity. Sweden has many immigrants from LMIC countries, but the disease burden of relevant comorbidities in Sweden differs only slightly from what is found in the other Nordic countries.

Timing in the initial phase of the pandemic was critical. In late February 2020 (week 9) hundreds of people returned from ski holidays in Austria and Italy, where hotels in Ischgl turned out to be virus hotspots. Until week 12 when Swedish authorities started advising the elderly to isolate, etc (see below) there were 3 weeks of uninhibited virus spread that already in week 12 led to excess mortality in Sweden. The other Nordic countries had earlier ski holidays (weeks 7-8) and fewer persons traveled for downhill skiing in the Alps.

## Were Swedish COVID-19 Policies Different?

The Swedish policy response to the pandemic came a little later and with much less stringency. The Oxford Government Response Tracker^21^ has developed the *Stringency Index* measuring how forceful government measures were implemented. On April 1—2 weeks after mortality started to increase in Sweden—the index was 85 in Spain, 79 in Norway and the United Kingdom, 72 in Denmark, 68 in Finland, and only 59 in Sweden. It is worth noticing that Spain and the United Kingdom had strong interventions, but the result was still not encouraging.^22^

Social distancing measures were implemented early in almost all European countries, but with different levels of stringency. Some adopted formal stay-at-home orders, but not in any of the Nordic countries. Closure of public spaces such as nonessential stores, bars, and restaurants was enforced in all countries except Sweden. Banning gatherings larger than 50 persons was introduced in all countries. All countries also closed secondary schools, universities, and all—except Sweden—closed primary schools and daycare (see Table 3). Swedish authorities argued that the parents were needed in health and social services. Sweden, like other countries, installed a ban to visit patients in nursing homes. But the general policy focus in all countries was much more on hospital and intensive care unit capacity than on protecting patients in elderly care and nursing homes.

**Table 3.** Protective Policies in Sweden and Five Comparable Countries During the First Wave of COVID-19 Pandemic.

	Denmark	Finland	Norway	Sweden	Spain	United Kingdom
Closed prim/sec schools (days)	30 of 63	57 of 89^a^	46 of 64	0 of >90	>90 of >90	69 of 83
Closure of public places (days)	33	74	54	0	50	64
Use of mask in closed env.	Recommended	Recommended	Not recommended	Not recommended	Compulsory	Recommended
Travel restrictions	Full	Selective	Full	Selective	Full	Selective

Source: OECD 2020.^7^

^a^ >90 days means that education was closed until the summer holiday.

Following the gradual easing of confinement measures, mask wearing was made compulsory in closed public areas including public transport in most European countries, but not in Sweden. Sweden was also slow in expanding test capacity. A month after each country reached a mortality rate >1 per 100 000 Denmark reported 250 tests per 100 000, the United Kingdom 78, Norway 55, and Sweden only 35.^7^

Sweden's excess mortality compared to the other Nordic countries was, as mentioned, concentrated to the elderly (Table 1), and nearly half the deaths occurred among nursing home clients. That raises the question whether Swedish care of the elderly differs in some important respects. The Nordic countries are all long known for tax-financed and publicly produced universal welfare services including schools, health, and social care. In the last 30 years, Sweden has privatized the still tax-financed public services and opened them up to for-profit enterprises.^23–25^ In the early 1990s, 2% of the public service employment in Sweden was in private nonprofit services and 2% was in for profit. In 2016, it was still only 3% nonprofit, but for profit had increased to 19%.^26^ In Stockholm, the for-profit proportion is 3 times as high. The internal quasimarkets created in Sweden in the 1990s to enable “consumer choice” have strengthened the for-profit welfare sector. While nonprofit organizations had difficulties in raising the necessary funds to join the state-funded contracts, the quasimarkets have attracted private investments and venture capital, even from abroad. In the social care of the elderly people, the for-profit activity covers more than 25%. In 2015, two companies (Ambea and Attendo) ran nearly half of all private residential care for older people in Sweden.^27^ The Swedish development has many parallels in the United Kingdom, but not in the other Nordic countries.

A recent review^5^ studied what characterizes elderly care and nursing homes with high COVID-19 incidence or mortality. They found that lack of access to personal protection equipment, lack of testing, less qualified staff and no nurses in the staff, bigger size of nursing homes, staff working part-time parallel in different care homes, and for-profit ownership were all important aspects. There are variations between Nordic countries in some of these dimensions, but they can hardly explain the higher mortality in Sweden. High work intensity and lack of support from leadership in nursing homes seem, however, to be a bigger problem in Sweden. That difference might be related to for-profit ownership and contribute to higher mortality.^5^

The impact of for-profit ownership of elderly care has been studied, but the results are mixed. Studies indicate that private care providers emphasize service aspects rather than structural prerequisites for good care such as a high staff/patient ratio.^28,29^ Although the principles of care as a social right are unchanged, the increasing for-profit element and free choice changes the ethos of the welfare state. The principles of universalism, inclusiveness, and equality are threatened by the logic of profit making and free choice.

The Swedish Inspectorate of Health and Social Services^30^ has evaluated how care homes for the elderly handled the COVID-19 pandemic and found several points to criticize. The access to primary health care was limited and 20% of COVID cases were not offered any medical examination. An intensive discussion on policy options and responsibilities has been the result.^31^ A government commission was appointed to analyze causes of the high COVID-19 mortality among the elderly in Sweden. Their report was published in December 2020 and they conclude that it was an explicit priority of the government to protect the elderly living in care homes, but the implemented policy failed in doing so.^32^ They suggest a broad range of initiatives, including better integration of medical and social care, improved working conditions, staffing and competence at nursing homes, and improved access to medical care.

## Why Were the Swedish Pandemic Policies Different?

When the pandemic took off in early March 2020, the evidence on what were the most effective interventions against the new virus was still scarce, and many senior epidemiologists in the Nordic countries suggested a less rigorous strategy without any lockdown, etc. The governments in Denmark and Norway made, however, the political decision to enforce a range of stringent measures including closure of schools and public places. At the same time, the Swedish Public Health Agency took a different and softer path by recommending that people aged 70+ limited their social contacts, and people were urged not to travel abroad. Personal responsibility rather than legislation was emphasized, and employers were asked to take measures so that employees could keep social distancing.

While it, in Denmark, was the prime minister who led the weekly press conferences, together with chiefs of police, it was in Sweden the chief epidemiologist of the public health agency who was in charge. It made it possible to convey to the Swedish public the scientific uncertainties of the interventions recommended and relied on trust between authorities and the population. There was a stark contrast between the very independent role played by the public health agency in Sweden and the more heavy-handed approach of the governments in other countries.

Sweden has a long tradition of independent government agencies. There is a dual system of public administration dating back to the early 18th century that differs from other European countries. The government agencies are subordinated the government, but they are at the same time quite independent of the government. Sweden does not allow ministerial rule, instead the government has to give directives collectively. The constitution restricts the government from influencing the agencies in matters concerning exercise of public authority against an individual, or a municipality, or influencing the adjudication process. Outside this limited area of application, it is allowed for the government to influence the agencies.^33,34^ Where exactly that limit is drawn is however under debate and has clearly been fluctuating in recent years. During the pandemic, the public health agency wanted and was given an unusually independent role.

In Figure 1, we illustrated that the policies to protect the population from the virus have unequal effects on employment and the economy (4). Even if the public health actions in Sweden were less stringent, the effect on the economic activity was not (Table 1). The public health policies have differed across the Nordic countries, but the social policies to protect the population against the socioeconomic effects have not.^35^ They have generally been rather extensive and generous which has limited the economic impact compared to the United Kingdom and Spain.

In summary, Sweden had during the first wave of the pandemic an excess mortality among the elderly. Higher levels of exposure to the virus due to an unfortunate timing of ski holidays, more relaxed control measures, and an elderly care system unprepared to protect the fragile elderly played a major role. Difference in susceptibility or comorbidity may not be important for understanding the country’s differences, but they are relevant for the sociodemographic inequalities within each country. Survival rates have improved, but the excess mortality in Sweden and in an increasing number of other European countries has continued into the second wave in November-December 2020.^2^ Denmark, Norway, and Finland now look increasingly as the outliers.
